# Relationship between Indices of Vascular Function and Presence of Overt Cardiovascular Disease among Persons with Poorly Controlled Type 2 Diabetes

**DOI:** 10.3390/jcdd8120185

**Published:** 2021-12-14

**Authors:** Sofia Antoniou, Katerina K. Naka, Marios Papadakis, Aris Bechlioulis, Dimitrios Makriyiannis, Agathocles Tsatsoulis, Lampros K. Michalis, Stelios Tigas

**Affiliations:** 1 Department of Endocrinology, University of Ioannina, 45110 Ioannina, Greece; sofia.antoniou3810@gmail.com (S.A.); atsatsou@uoi.gr (A.T.); 2 2nd Department of Cardiology and Michaelidion Cardiac Center, University of Ioannina, 45110 Ioannina, Greece; drkknaka@gmail.com (K.K.N.); drbech1979@gmail.com (A.B.); lamprosmihalis@uoi.gr (L.K.M.); 3 Department of Surgery, Helios Clinic, University Hospital Witten-Herdecke, 42283 Wuppertal, Germany; marios_papadakis@yahoo.gr; 4 Department of Medicine, Hatzikosta General Hospital, 45445 Ioannina, Greece; md02798@yahoo.gr

**Keywords:** diabetes mellitus, cardiovascular disease, subclinical atherosclerosis, endothelial function, carotid intima–media thickness

## Abstract

The aim of this study was to assess the factors associated with impaired vascular function in patients with poorly controlled type 2 diabetes (DM2) with and without overt cardiovascular disease (CVD). Ninety-five patients with DM2 and poor glycemic control were recruited and divided into two groups: Group 1, with known CVD (*n* = 38), and Group 2, without CVD (*n* = 57). Patients in Group 2 were further subdivided into those with short (<5 years, group 2b) and long (>5 years, group 2a) diabetes duration. Subclinical markers of atherosclerosis were assessed. Glycemic control was similar in the two groups (HbA1c: 9.2% (1.5) vs. 9.4% (1.8), *p* = 0.44). In Group 1, lower FMD (3.13 (2.16)% vs. 4.7 (3.4)%, *p* < 0.05) and higher cIMT (1.09 (0.3) mm vs. 0.96 (0.2) mm, *p* < 0.05) was seen compared with Group 2, whereas PWV was similar (12.1 (3.4) vs. 11.3 (3.0) m/s, *p* = 0.10). Patients in Group 2b had significantly lower PWV and cIMT and higher FMD compared to Group 1 (*p* < 0.05). Among patients with poorly controlled T2D, more pronounced vascular dysfunction was present in those with overt macrovascular disease. In patients with T2D without known CVD, vascular dysfunction was associated with disease duration. The use of vascular indices for cardiovascular risk stratification in patients with T2D requires further study.

## 1. Introduction

Atherosclerotic cardiovascular disease (ASCVD) remains the main cause of death in patients with type 2 diabetes mellitus (DM2) and results in significant morbidity and increasing health care costs [1]. Many patients are in fact already predisposed to significant cardiovascular risk even at the prediabetic stage [2]. Moreover, they often remain asymptomatic and when they develop clinically evident CVD, their prognosis and outcomes are worse compared to individuals with CVD but without DM2 [3,4].

Large epidemiological studies have established the role of hyperglycemia as a risk factor for the development of both microvascular and macrovascular complications [5,6]. Hyperglycemia alters the protective mechanisms of the endothelium and leads to inflammatory damage of the endothelial wall, increased permeability, and a reduction in antiatherogenic vasodilators such as nitric oxide [7]. Endothelial dysfunction has been previously reported in patients with DM2 as well as prediabetes compared to healthy subjects [8,9,10,11], while comorbidities such as hypertension and dyslipidemia seem to have a synergistic effect, leading to accelerated atherogenesis [12,13,14].

In recent decades, several non-invasive techniques have been developed as research tools to assess vascular dysfunction, potentially enabling the identification of high-risk patients at an early, subclinical stage of vascular disease. The use of non-invasive markers of subclinical atherosclerosis may provide useful, early, and cost-effective information on vascular properties and the possible effects of pharmaceutical or non-pharmaceutical interventions.

The aim of the present study was to assess vascular dysfunction by using brachial artery flow-mediated dilation (FMD), carotid–femoral pulse wave velocity (cfPWV), central augmentation index (AIx), large (C1) and small (C2) artery compliance, carotid intima–media thickness (cIMT), and ankle–brachial index (ABI) in poorly controlled DM2 patients with and without overt CVD as well as to examine the role of DM2 duration.

## 2. Materials and Methods

Patients with DM2 were prospectively recruited from the Department of Endocrinology at Ioannina University Hospital and the Diabetes Outpatient Clinic at Chatzikosta General Hospital, Ioannina between July 2011 and December 2014. Eligible subjects were patients with DM2 and poor glycemic control, as defined by levels of HbA1c of ≥7.5%. Disease duration was defined as the time interval between age at diagnosis and study entry, as recorded in patients’ clinical records. The presence of established macrovascular complications was evaluated on the basis of the patients’ past medical history obtained by experienced cardiologists and endocrinologists. The study subjects were divided into two groups: Group 1, patients with DM2 and known CVD (history of any coronary artery, cerebrovascular or peripheral vascular disease), and Group 2, patients without CVD. The duration of diabetes in all Group 1 patients was more than 5 years, whereas patients in Group 2 were further subdivided into those with short (<5 years, Group 2a) and long (>5 years, Group 2b) diabetes duration.

Informed consent was obtained from all participants. Patients with chronic liver disease or severe heart failure, those on treatment for cancer or on glucocorticoids, subjects with a history of drug or alcohol abuse, or subjects suffering from severe psychiatric disorders were excluded. The study was approved by the local ethics committee and complied with international ethical norms and standards according to the Helsinki Declaration. Data on cardiovascular risk factors were collected from all patients.

Hypertension was defined as blood pressure >140/90 mmHg or use of antihypertensive medication. Hypercholesterolemia was defined as a level of total cholesterol >200 mg/dL or use of lipid-lowering agents. Patients who were smoking at the time of study or had stopped smoking during the last 12 months prior to the study were defined as current smokers. Exercise was defined as brisk walking for at least 30 min, 3 times weekly. Presence of overt macrovascular disease was evaluated by experienced physicians based on review of medical records, history and physical examination.

FMD was assessed at the brachial artery by using a 5–12 MHz linear transducer of an Echo-Doppler ultrasound (Vivid-I, General Electric, IL, USA), at baseline and at 5 min after cuff inflation at 250 mm Hg, as previously described [15,16]. The measurement of the common carotid artery IMT was assessed by using B-Mode ultrasound with semi-automated edge detection software (Vivid-I, General Electric, Chicago, IL, USA). Both the average and maximal cIMT measurement at a length of at least 1 cm on the distal wall of the common carotid artery were recorded according to recent consensus of the American Society of Echocardiography [16,17]. Applanation tonometry (Sphygmocor system, AtCor Medical, Sydney, Australia) was used to non-invasively assess carotid femoral pulse wave velocity (cfPWV). Transmission time was calculated with a special system software that uses the R-wave of an electrocardiogram as a reference frame and a given distance from stable anatomical positions (suprasternal notch–femoral artery). The Augmentation Index (AIx@75) was calculated using the latter systolic pressure peak of the obtained waveform and was normalized for a heart rate of 75 bpm, as previously described [18,19]. The HDI Pulse Wave CR2000 was used to assess small and large artery compliance, by obtaining non-invasively radial arterial measurements using an oscillometric technique, as previously described [20]. ABI was calculated as the ratio of average systolic blood pressure at the ankle of each leg divided by the average systolic blood pressure of the arm using a portable Doppler device.

HbA1c levels were measured with a point-of-care analyzer (DCA Analyzer, Siemens 2007), that requires a small blood sample (1 μL) and provides reliable results within 6 min. Blood samples were used to measure other important parameters as well, such as serum total cholesterol, high-density lipoprotein (HDL), triglycerides, urine, and creatinine. Low-density lipoprotein component (LDL–C) was calculated using the Friedwald formula (LDL-C = Total Chol-HDLc-TRG/5) [21]. Elevated LDL levels were defined as >130 mg/dL and high triglyceride levels were defined as >150 mg/dL. Body mass index (BMI) was calculated as weight/height^2^ (kg/m^2^). The minimum waist measurements between the pelvic brim and the costal margin and the maximum hip measurement at the level of the greater trochanters were used to calculate the waist to hip ratio.

Normal distribution was determined using histogram plots, box plots, and the Shapiro–Wilk test. All continuous variables had a normal distribution and are presented in mean ± standard deviation (SD) form. Categorical variables were compared using the two-sided Fischer’s exact test and the Chi-Square test. Correlation analysis was performed by using Pearson’s correlation to determine r and two-tailed *p* values. The two-tailed student t-test was employed to compare continuous variables between the two groups. Further subgroup analysis was performed using a one-way ANOVA test or Kruskal–Wallis test (in the case of age, diabetes duration, HDL, and ABI) for continuous variables and chi-square test for dichotomous variables. In the case of a significant ANOVA, post hoc analysis was performed using Bonferroni test. A *p*-value of <0.05 was considered statistically significant. Data analyses were performed using SPSS Version 16.0.

## 3. Results

One hundred and eight patients were prospectively recruited over the study period. Thirteen patients did not meet the inclusion criteria and were therefore excluded from the study. The total study population consisted of 95 DM2 patients: 53 males (56%) and 42 females (44%).

Thirty-eight patients had established CVD (Group 1), whereas 57 had no known history or symptoms suggestive of CVD (Group 2). Mean age was 65 (9) and 62 (10), respectively (*p* = 0.16). The mean duration of diabetes was similar between the groups (16 (10) vs. 13 (9) years, *p* = 0.14), although 14 patients of Group 2 had a shorter disease duration of <5 years. The impact of diabetes on the vasculature of this subgroup was analyzed separately. Glycemic control was similar between the two groups (mean HbA1c 9.2 (1.5) vs. 9.4 (1.8)%, *p* = 0.44). Patients with CVD had a higher prevalence of hypertension and hypercholesterolemia (100% vs. 74% and 87% vs. 54%, *p* < 0.05 for both), as well as lower HDL levels (43 (9) mg/dL vs. 50 (13) mg/dL, *p* < 0.05). This could be partly explained by the more frequent use of statin treatment and by lifestyle modification in patients with established CVD. The demographic, clinical, biometric, and laboratory parameters of all study participants are summarized in Table 1.

All continuous variables are normally distributed and therefore expressed as the mean (standard deviation) form. Categorical variables are expressed in terms of absolute (n) and relative (%) frequencies. Statistical significance: *p*-value < 0.05. NS = not significant.

Patients with CVD were treated more often with antihypertensive medications, insulin, beta-blockers, antiplatelet drugs, and statins (*p* < 0.05) (Table 2). Use of metformin was similar between the two groups, whereas use of sulfonylureas was more frequent among patients without CVD (40% vs. 24%) (Table 2).

Hemodynamic parameters and markers of subclinical atherosclerosis are presented in Table 3. Systolic and diastolic blood pressure and heart rate were comparable in both groups. Patients with CVD had higher cIMT (0.82 (0.2) vs. 0.69 (0.2) mm, *p* < 0.05) and lower FMD compared to those without known CVD (3.13 (2.6) vs. 4.7 (3.4)%, *p* < 0.05), whereas PWV was similar in both groups (12.1 (3.4) vs. 11.3 (3), *p* = 0.1). HbA1c did not correlate with any markers of subclinical atherosclerosis, whereas we observed a correlation between age and PWV, as expected.

The second group was divided into two subgroups, based on diabetes duration. In 43 patients, the disease had been diagnosed >5 years prior to enrollment (Group 2a), whereas 14 patients had a shorter disease duration (diagnosed <5 years prior to enrollment) (Group 2b). A comparison of various parameters among the three groups using one-way ANOVA or the Kruskal–Wallis test demonstrated statistically significant differences in diabetes duration, HDL levels, cfPWV, FMD, cIMTaver, and cIMTmax (Table 4). Post hoc analysis revealed that Groups 2a,b had significantly higher HDL levels compared with Group 1. Vascular function indices differed significantly between Groups 1 and 2b; patients with DM2 of short duration and no CVD had significantly higher FMD, and lower cfPWV and cIMT compared with patients with CVD (*p* < 0.05 for all in post hoc analysis). No other significant correlations were observed.

## 4. Discussion

This study demonstrated that in poorly controlled patients with DM2, the presence of macrovascular disease was associated with more pronounced vascular dysfunction (lower FMD) and higher cIMT. Patients with shorter disease duration (less than 5 years since diagnosis) and no CVD had significantly higher FMD, and lower PWV and cIMT compared to those with CVD and longer diabetes duration. The use of non-invasive markers of subclinical atherosclerosis may potentially provide a tool to enable more accurate cardiovascular risk stratification in patients with T2D.

FMD expresses the percentage of increase in the brachial artery diameter after induced hyperemia, increasing NO release. FMD offers a noninvasive endothelial function assessment in conduit arteries, which are affected by the development of atherosclerotic disease. Since its introduction in the early 1990s by Patel et al. [22], the method has been used as a surrogate end point in many clinical trials. A meta-analysis concluded that FMD varies from 0.20 to 19.2% in healthy populations, while in subjects with CHD and DM2 FMD ranged from −1.3 to 14% and from 0.75 to 12%, respectively [23]. Significant associations were previously found between FMD and diabetes duration by our group [19]. Although FMD-detectable endothelial dysfunction has been reported even in newly diagnosed patients [24], the synergistic effect of CHD and DM2 on FMD has been previously questioned. Simova et al. showed that the presence of DM2 was associated with differences in FMD in patients with different degrees of coronary artery stenosis [25]. Ito et al. showed that when compared with healthy subjects, the presence of DM2, CHD, or both resulted in lower FMD levels but found no clear difference in the FMD between subjects with DM2 and CHD versus those with DM2 and no CHD [26]. Zhang XG et al. studied patients with DM2 and demonstrated that blood glucose fluctuations affect the inflammatory response and possibly induce CHD in these patients, as reflected by the impairment of FMD [27].

Carotid intima media thickness measurement (cIMT) is another non-invasive method used to identify subclinical vascular carotid disease. cIMT reflects structural rather than functional changes in the arterial wall [17,28,29]. Normally, values increase with age; however, evidence of the acceleration of cIMT progression has been shown in the presence of metabolic disorders such as diabetes; in a meta-analysis including 24,111 patients from 24 studies, Brohall et al. showed an additional mean cIMT increase of 0.13 mm in DM2 patients, suggesting a higher relative risk for cardiovascular events [30]. Tasic et al. reported that the presence of hypertension in subjects with DM2 is associated with a higher incidence of asymptomatic carotid artery disease and left ventricular hypertrophy as measured by cIMT and left ventricular mass in echocardiography [31]. Furthermore, the Insulin Resistance Atherosclerosis Study showed that subjects with DM2 and coronary artery disease had greater cIMT, compared with those without DM2 [32]. In accordance with these findings, we showed that in patients with CVD and DM2, the mean cIMT was significantly higher compared to those with DM2 but no CVD (0.82 vs. 0.7, respectively, *p* < 0.05). An even larger difference was observed when DM2 patients with known CVD and long duration of the disease were compared with those with no history of CVD and shorter (<5 years since diagnosis) disease duration (0.57 mm, *p* < 0.05).

Pulse Wave Velocity (PWV) is a useful tool commonly used to assess arterial stiffness, an index that reflects functional wall properties and expresses the propagation speed of the pulsation along the arterial tree [33,34,35]. It normally increases with age; this process is however accelerated by the presence of CVD risk factors such as hypertension or DM2 [36,37,38]. Previous studies have shown that PWV is higher among patients with DM2 and can be observed even prior to the onset of the disease, in patients with impaired glucose tolerance [39,40,41]. In an observational study with 2080 subjects, Tomiyama et al. showed that raised blood pressure and plasma glucose levels, even below those defining hypertension and diabetes, may synergistically lead to the progression of arterial stiffening [42]. In the same direction, Bruno et al. compared hypertensive patients with and without DM2 and showed that the coexistence of hypertension and DM2 worsens arterial stiffness, as indicated by higher PWV and lower FMD [43]. In our study, PWV was not significantly higher in subjects with DM2 and CVD, compared with those without CVD, probably reflecting the early onset of arterial stiffness in DM2 even in the absence of overt CVD.

Our study has several limitations that should be considered. First, the number of participants studied was relatively small; more extensive studies are needed to confirm our results. Secondly, subjects with a disease duration of less than five years were considered as newly diagnosed, although for many patients, the exact time of initial diagnosis remains unclear. Moreover, patients with short diabetes duration were also younger, which could potentially bias our results. Markers of insulin resistance and inflammation were not assessed in the present study, which is another potential limitation.

Finally, it should be noted that the present study was designed and conducted at a time when newer antidiabetic agents with proven CVD benefits such as glucagon-like peptide-1 receptor agonists (GLP-1 RAs) and sodium–glucose cotransporter-2 (SGLT2) inhibitors were not widely available. Taking into consideration recent evidence that SGLT2 inhibitors may significantly improve FMD, while GLP-1 RAs may reduce PWV [44], it is impossible to know whether the observed differences in the vascular indices of DM2 patients with and without CVD would still apply in patients treated with these newer antidiabetic medications. We recently showed that in patients with long-standing diabetes treated with insulin and older antidiabetic agents, endothelial function, elasticity, or arterial stiffness of large arteries failed to improve and cIMT deteriorated despite short-term aggressive glycemic control [16]. On the other hand, emerging evidence suggests that intensive treatment with incretin-based agents may result in improvements in FMD, PWV, and markers of oxidative stress [45].

Previous studies suggested that DM2 is a coronary heart disease (CHD) risk equivalent, leading to recommendations and health policies addressing the need for aggressive CV risk management and pharmacotherapy [46]. More recently, however, it has been recognized that not all DM2 patients carry the same CVD risk, emphasizing the need for a more individualized approach based on each subject’s cardiovascular risk [47]. Of note, in patients with longer disease duration, DM2 appears to be a CHD equivalent, whereas this is not necessarily the case for those at an earlier stage of the disease [48,49,50]. Apart from glycemic control, cardiovascular risk in patients with T2D is influenced by a number of factors such as obesity, hypertension, pro-atherogenic lipids, and the presence of insulin resistance and low-grade chronic inflammation [51]. Current international guidelines emphasize the importance of an individualized approach to the treatment of patients with T2D; amongst other factors, the presence of ASCVD and the duration of the disease are important in order to guide clinical decisions on (a) the choice of antihyperglycemic therapy and (b) the glycemic treatment target [1]. For example, treatment with GLP-1 RAs and/or SGLT2 inhibitors is recommended to those with DM2 and established ASCVD or multiple risk factors, independent of their level of glycemic control [1,52,53,54]. Taking into consideration the recent change in clinical practice and the promising results of newer antidiabetic agents in terms of both subclinical atherosclerosis markers and clinical endpoints, it would be of great interest to further study the use of such vascular indices for early detection and better risk stratification to prevent macrovascular complications in the DM2 patient population [55].

## 5. Conclusions

We conclude that in patients with poorly controlled DM2, the presence of macrovascular disease is associated with more pronounced vascular dysfunction, as assessed with non-invasive markers of subclinical atherosclerosis. Furthermore, patients with relatively short duration of diabetes (<5 years since diagnosis) had a significantly better vascular profile. Further prospective studies are needed to explore the potential use of vascular indices for cardiovascular risk stratification in patients with DM2 with or without CVD.

## Figures and Tables

**Table 1:** Characteristics of the study population.

Category	Parameter	DM2 with CVD (Group 1, *n* = 38)	DM2 without CVD (Group 2, *n* = 57)	*p*-Value
Demographics	Age (years)	65 (9)	62 (9.6)	0.163
	Gender (male)	23 (61%)	30 (53%)	0.53
	Diabetes duration (years)	15.6 (9.6)	12.8 (8.9)	0.138
Comorbidity	Hypertension	38 (100%)	42 (74%)	*p* < 0.05
	Hypercholesterolemia	33 (87%)	31 (54%)	*p* < 0.05
	Smoking	18 (47%)	19 (33%)	0.2
	Exercise	24 (63%)	37 (65%)	1
Family history	Family history of CVD	11 (29%)	10 (18%)	0.214
	Family history of DM2	17 (45%)	32 (56%)	0.301
Biometrics	BMI (kg/m^2^)	29.5 (5.7)	30.7 (7)	0.37
	Hip (cm)	106.2 (10.8)	106.1 (14.0)	0.974
	Waist (cm)	105.1 (13.3)	103.4 (14.0)	0.56
	Waist-to-hip ratio	0.99 (0.07)	0.96 (0.08)	0.068
Laboratory measurements	HbA1c (%)	9.2 (1.5)	9.4 (1.8)	0.444
	Chol (mg/dL)	188 (51)	197 (39)	0.314
	HDL (mg/dL)	43 (9)	50 (13)	*p* < 0.05
	LDL (mg/dL)	111 (45)	114 (32)	0.657
	TRG (mg/dL)	178 (125)	165 (129)	0.636
	Creatinine (mg/dL)	1.1 (0.29)	1.0 (0.29)	0.101

Abbreviations. BMI: body mass index; HbA1c, glycated hemoglobin; HDL, high-density lipoprotein; LDL, low-density lipoprotein; TRG: triglycerides.

**Table 2:** Medications used in subjects with DM2 and CVD (Group 1) or without CVD (Group 2).

Drugs	DM2 with CVD (Group 1, *n* = 38)	DM2 without CVD (Group 2, *n* = 57)	*p*-Value
Insulin, n (%)	22 (58%)	17 (30%)	*p* < 0.05
Sulfonylureas, n (%)	9 (24%)	23 (40%)	0.122
Glimepiride, n (%)	6 (16%)	10 (18%)	1
Metformin, n (%)	27 (71%)	40 (70%)	1
DPP4-inhibitors, n (%)	8 (21%)	13 (23%)	1
Pioglitazone, n (%)	1 (3%)	4 (7%)	0.645
Meglitidines, n (%)	1 (3%)	2 (4%)	1
Alpha-glucosidase inhibitors, n (%)	0	1 (2%)	1
Calcium channel blockers, n (%)	10 (26%)	13 (23%)	0.808
ACE inhibitors, n (%)	10 (26%)	11 (19%)	0.457
Beta-blockers, n (%)	27 (71%)	12 (21%)	*p* < 0.05
ARBs, angiotensin II receptor blockers, n (%)	18 (47%)	26 (46%)	1
Diuretics, n (%)	17 (45%)	16 (28%)	0.124
Antiplatelets, n (%)	27 (71%)	10 (18%)	*p* < 0.05
Statins, n (%)	32 (84%)	24 (42%)	*p* < 0.05
Fibrates, n (%)	7 (18%)	4 (7%)	0.109

Abbreviations. ACE, angiotensin-converting enzyme; ARBs, angiotensin II receptor blockers; DPP4, dipeptidyl peptidase 4. Values expressed as absolute numbers (n) and relative frequencies (%). Statistical significance: *p*-value < 0.05. NS = not significant.

**Table 3:** Basic hemodynamic parameters and markers of subclinical atherosclerosis in Groups 1 and 2.

Parameter	DM2 with CVD (Group 1, *n* = 38)	DM2 without CVD (Group 2, *n* = 57)	*p*-Value
cfPWV, m/s	12.1 (3.4)	11.3 (3)	0.099
HR, bpm	67 (11.3)	69 (11.5)	0.34
SBP, mmHg	136.2 (20.1)	130.4 (17.5)	0.16
DBP, mmHg	75.8 (10.3)	74.5 (12.8)	0.59
C1, mL/mmHg × 100	14.3 (5.7)	13.9 (5.6)	0.74
C2, mL/mmHg × 10	4 (1.59)	4.1 (2.1)	0.86
ABI	0.91 (0.13)	0.95 (0.1)	0.15
FMD, %	3.13 (2.6)	4.7 (3.4)	*p* < 0.05
cIMTaver, mm	0.82 (0.24)	0.69 (0.19)	*p* < 0.05
cIMTmax, mm	1.09 (0.29)	0.96 (0.24)	*p* < 0.05

Abbreviations: ABI, ankle–brachial index; AIx@75, augmentation index; AcfPWV, carotid–femoral pulse wave velocity; cIMT, carotid intima–media thickness; C1, large artery elasticity index; C2, small artery elasticity index; DBP, diastolic blood pressure; HR, heart rate; FMD, flow-mediated dilation. Values expressed as mean (standard deviation) form. Statistical significance: *p*-value < 0.05.

**Table 4:** Comparison of various variables between subgroups of patients according to the presence of cardiovascular disease and duration of diabetes.

Parameter	Group 1 DM2 with CVD *n* = 38	Group 2a DM2 without CVD >5 Years Since Diagnosis *n* = 43	Group 2b DM2 without CVD <5 Years Since Diagnosis *n* = 14	*p*-Value
Age, years	65 (9)	64 (8)	57 (12.5)	*p* = 0.18
Diabetes duration, years	15.6 (9.6)	16.2 (7.5)	2.3 (1.7) *^,†^	*p* < 0.05
HDL, mg/dL	43 (9)	48.9 (13.5) *	54.5 (9.5) *	*p* < 0.05
Creatinine	1.1 (0.29)	1.0 (0.31)	0.9 (0.13)	*p* = 0.06
SBP, mmHg	136 (20)	133 (17)	123 (17)	*p* = 0.07
cfPWV, m/s	12.1 (3.4)	11.8 (3.1)	9.9 (1.98) *	*p* = 0.04
C1, mL/mmHg × 100	14.3 (5.7)	13.9 (6.0)	13.6 (4.3)	*p* = 0.93
C2, mL/mmHg × 10	4.0 (1.59)	4.1 (2.3)	3.8 (1.6)	*p* = 0.81
ABI	0.91 (0.13)	0.94 (0.09)	0.97 (0.05)	*p* = 0.39
FMD, %	3.1 (2.6)	4.3 (3.2)	5.9 (3.8) *	*p* = 0.02
cIMTaver, mm	0.82 (0.24)	0.73 (0.19) *	0.57 (0.13) *	*p* < 0.05
cIMTmax, mm	1.09 (0.29)	1.00 (0.25)	0.83 (0.17) *	*p* < 0.05

* *p* < 0.05 compared with Group 1 in post hoc analysis, ^†^ *p* < 0.05 compared with Group 2a in post hoc analysis. Abbreviations: ABI, ankle–brachial index; cfPWV, carotid–femoral pulse wave velocity; cIMT, carotid intima–media thickness; C1, large artery elasticity index; C2, small artery elasticity index; FMD, flow-mediated dilation; HDL, high-density lipoprotein cholesterol; SBP, systolic blood pressure.

## Data Availability

Data are available from the corresponding author upon reasonable request.
